# Avoiding the “déjà-variable” phenomenon: social psychology needs more guides to constructs

**DOI:** 10.3389/fpsyg.2014.00052

**Published:** 2014-01-31

**Authors:** Martin S. Hagger

**Affiliations:** Health Psychology and Behavioural Medicine Research Group, Laboratory of Self-Regulation, Faculty of Health Sciences, School of Psychology and Speech Pathology, Curtin UniversityPerth, WA, Australia

**Keywords:** constructs, integrative frameworks, research synthesis, systematic reviews, intention

As a journal editor, I am frequently asked what constitutes an exceptional research article (Hagger, 2012). I usually respond by recommending Skinner's (1996) seminal guide to constructs of control as a prototypical example. When I was a doctoral student Skinner's article was extremely influential to my work. It not only helped me make sense of the myriad of constructs and terms used to describe and define the *control* construct, but also how I approached other constructs in social psychology. Skinner's guide was ahead of its time and her systematic approach to classifying and synthesizing constructs is supremely relevant today. Skinner identified the diversity in terminology, definition, and content of constructs related to control in social psychology, conducted an integrative review of theories incorporating control-related constructs, and developed a taxonomy and theoretical model to characterize them. Her guide was innovative for three reasons: (1) she recognized the richness in control-related constructs in social psychology but also the substantial redundancy and poor consensus; (2) she focused on systematically reducing redundancy and arriving at a core set of definitions and constructs; (3) she developed a theoretical basis for her integration. The proliferation of systematic reviews and syntheses in social psychology and other psychological disciplines illustrates that Skinner's guide was a precursor of things to come, but also illustrates that many syntheses of research findings are somewhat premature as they do not first systematically identify the commonalities and diversity in the constructs involved (Hagger and Chatzisarantis, 2009). I argue that social psychology needs more integrative frameworks like Skinner's to identify consistency in terminology, definition, and content of social psychological constructs. This will assist researchers to synthesize and integrate research findings and effects involving social psychological constructs.

There have been many occasions when I have felt confronted with an uncanny sense of déjà-vu when reading social psychologists' descriptions of constructs they had developed. I call this the *déjà-variable* phenomenon; the feeling that one has seen a variable with the same definition and content before only referred to by a different term. And if not precisely identical, one can recognize considerable overlap and redundancy in the definition of variables making it difficult to establish whether constructs with different terms are appreciably different in content. This generates problems, particularly for the uninitiated, when it comes to making sense of research findings and hinders the progress of psychological science. Skinner notes that the use of constructs with “… many different names for the same construct has interfered with the accumulation of research findings. Findings about a construct under one label may never be integrated with findings about the same construct under different labels” (p. 550). Similarly, Block (1995), after Kelley (1927), calls this the “jangle” fallacy which “waste[s] scientific time” (p. 210). Researchers attempting to make sense of effects involving a specific construct in social psychology may neglect entire literatures because they fail to recognize constructs consistent with their definition but labeled with a different term. This presents considerable challenges for those engaged synthesizing research findings, such as those compiling systematic reviews and meta-analyses, where coding of constructs and resolving variations or similarities in content of variables with differing terms is an essential part of the process. It also presents problems for researchers attempting to integrate and unify different theories where eliminating redundancy is an important goal (Hagger, 2009). Researchers conducting systematic reviews and meta-analyses or integrating theories must, therefore, be extremely diligent to avoid a “surface” approach to identifying relevant constructs in their analysis and conduct “deeper” analyses of content when searching for identical and overlapping constructs across the literature. Furthermore, constructs with a high degree of conceptual overlap and redundancy are also likely to be extremely difficult to distinguish empirically. For example, the inclusion of conceptually identical or similar variables in a statistical analysis is likely to raise problems such as multicolinearity. Researchers must take care when integrating constructs to ensure that they do not include constructs with identical or extremely similar content in their analyses.

Alongside the déjà-variable phenomenon there is the case where the same term is used for constructs with different definitions and content. This creates additional problems for social psychologists attempting to synthesize effects of constructs in a particular literature. As Skinner points out, “when the same term is used to refer to different constructs, reviewers may conclude that findings are inconsistent or even contradictory, when in fact it is definitions that are inconsistent and contradictory” (p. 550). This phenomenon has been previously labeled the “jingle” fallacy and Block (1995) warns that “… the unwary may consider [the constructs] interchangeable” (p. 209). Again, systematic reviewers need to be diligent when coding variables and focus on content as well as terminology when searching, identifying, including, and eliminating constructs in their attempts to distil effects across the literature. A good illustrative example is the construct of *intention*. Observing the diversity of constructs that have fallen under the term intention, and how this may lead to confusion when arriving at consensus among findings, suggests that a guide to the construct of intention would be timely and useful. As an example of the diversity among types of intention one need only turn to Meiland's (1970) seminal work which illustrates how intention has multiple definitions and content. Meiland demonstrated the necessity of identifying the core content of a construct (i.e., what it is and what is not) and advocated qualifying terms to signify additional content that distinguishes particular subtypes from the core; good recent examples include *continuation* intentions (Chatzisarantis et al., 2004) and *implementation* intentions (Gollwitzer, 1999; Hagger and Luszczynska, 2014).

Social psychologists need to adopt a similar approach to that of Skinner to arrive at a set of guides for constructs in social psychology. The guides will serve as invaluable summaries and compendiums of terms for social psychologists to use when operationalizing constructs in theories and models, developing measures, identifying patterns of effects, and developing explanations of processes and mechanisms. I also envisage these guides to be flexible, “living” documents that are constantly updated as theory and evidence for constructs evolves.

## Author contributions

Martin S. Hagger conceived the ideas presented in the article and drafted the article.
